# Rise of Marburg virus in Africa: a call for global preparedness

**DOI:** 10.1097/MS9.0000000000001257

**Published:** 2023-09-01

**Authors:** Olalekan J. Okesanya, Emery Manirambona, Noah O. Olaleke, Hisham A. Osumanu, Ayodeji A. Faniyi, Oumnia Bouaddi, Olatunji Gbolahan, Jose J. Lasala, Don E. Lucero-Prisno

**Affiliations:** a Department of Medical Laboratory Science, Neuropsychiatric Hospital, Aro, Abeokuta; b Department of Medicine and Surgery, University of Ilorin, Kwara; c Obafemi Awolowo University Teaching Hospital Complex, Ile-Ife; d Department of Medical Laboratory Science, Obafemi Awolowo University Teaching Hospitals Complex, Ile Ife, Osun State, Nigeria; e Department of Infectious Diseases, School of Allied Health Sciences, University for Development Studies, Ghana; f International School of Public Health, Mohammed VI University of Health and Sciences, Casablanca, Morocco; g College of Medicine and Health Sciences, University of Rwanda, Kigali, Rwanda; h College of Medicine, University of the Philippines, Manila, Philippines; i Department of Global Health and Development, London School of Hygiene and Tropical Medicine, London, United Kingdom

**Keywords:** Africa, Marburg virus, MARV, outbreaks

## Abstract

The Marburg virus disease (MVD) is caused by a rare RNA virus that can result in severe hemorrhagic fever in humans and other primates. The disease was first discovered in 1967 in Marburg Frankfurt in Germany and since then, sporadic cases have been reported in southeastern Africa. The Egyptian fruit bat is considered a reservoir for the virus, which can be transmitted through direct contact with infected bat or monkey tissue, bodily fluids, or contaminated objects. The Marburg virus disease shares clinical features with the Ebola virus disease, and there are no widely accepted vaccines or antiviral medications to treat it. The article provides an overview of Marburg virus (MARV) outbreaks in Africa, including the most recent outbreaks in Guinea, Ghana, Equatorial Guinea, and Tanzania. The authors discuss the recent outbreaks and the implications of the spread of MARV to Africa’s healthcare systems. The authors also present key recommendations for both multicountry and global preparedness efforts in order to better prevent and respond to future MARV outbreaks and other viruses with an epidemic potential.

## Background

The Marburg virus disease (MVD) is a severe and rare hemorrhagic fever caused by a unique RNA virus called the Marburg virus (MARV), which can affect both humans and nonhuman primates. MARV, a member of the Filoviridae family shares similarities with the Ebola virus^1^. The MARV consists of five strains and is classified as a biosafety level 4 agent due to its high fatality rate, direct person transmission, and lack of vaccines. Under an electron microscope, Marburg virion show pleomorphism, containing genomic RNA, large L protein, nucleoprotein, and virion proteins 30 and 35 within a lipoprotein unit membrane envelope. The virus has a stable infectivity at room temperature and its genome consists of linear, nonsegmented, negative-sense, single stranded RNA with a single overlapping region (VP30/VP24) with a length ranging from 800 to 14 000 nm^2,3^. MARV has seven structural proteins and elicits different antibodies compared to the Ebola virus. Although less well-known, MARV remains a significant viral pathogen^1^. In 1967, the virus was discovered during outbreaks of hemorrhagic fever in Marburg and Frankfurt in Germany, and in Yugoslavia and Belgrade. The outbreaks resulted in 31 cases and seven deaths. The virus was transmitted to individuals who were working with imported African green monkeys or their tissues^4^. Over the course of the years, MARV has been reported sporadically and isolated among residents and travelers in southeastern Africa. There have been several reported cases of MARV between 1998 and 2000 among young male workers in Durban, South Africa, and the Democratic Republic of Congo (DRC)^5^.

The Egyptian fruit bat ʻ*Rousettus aegyptiacus*ʼ is believed to be a reservoir for MARV, as several bat species are often hosts for the filovirus family according to previous research studies^6^. The virus can be transmitted directly from bats or monkeys to humans or from human to human through various routes including direct contact with infected bat shedding or monkey tissue, bodily fluids like blood, stool, urine, sweat, breast milk, or amniotic fluids, contaminated medical instruments or objects, and sexual contact with a recently recovered person with no active symptoms^4,7^. MARV has a similar pathogenesis to the Ebola virus and clinical features resembling tropical diseases. Its incubation period ranges from 2 to 21 days and it initially presents with fever, headache, muscle aches, and a rash^7^.

MARV continues to be a global health concern, with a case-fatality ratio of over 88%^8^ and periodic outbreaks in Central Africa. There were occurrences in Uganda in 2012 and 2014. The WHO stated that the possibility of a MARV outbreak spreading is high at the national level but low at the global level^1^. Currently, WHO is assisting the Ministry of Health of all affected country in enhancing various response pillars such as surveillance at entry points, laboratory capabilities, case management, risk communication, infection prevention, control, and community engagement^4^. In response to the emergence of the MARV in Africa, the CDC has issued travel advisories of varying levels 1 and 2 for affected countries but does not currently recommend international travel or trade restrictions. These precautionary alerts are influenced and justified by past experiences with comparable events^9^. MARV shares several clinical features and transmissibility comparable to the Ebola virus, requiring similar infection prevention and control measures including safe burials. Following the recent outbreaks of MARV in Ghana, Tanzania, and Equatorial Guinea, respectively, there is an increase of burden of this viral disease in Africa^10,11^. However, less is known about MARV maybe due to the absence of a recent large-scale outbreak like the West Africa Ebola outbreak^10^. This highlights the importance to address the challenges posed by MARV and infectious diseases in the African region. A better understanding of the factors that contribute to disease outbreaks, as well as the development of effective prevention and control strategies will significantly contribute to tackling the infection^12^. Therefore, our paper seeks to succinctly discuss the most recent outbreaks and implications of MARV in the African region, as well as provide key recommendations for both multinational and global preparedness to better manage, curb, and prevent unforeseen epidemics in the African continent.

## Updates on MARV outbreaks in Africa

The MARV has been responsible for many outbreaks since it was first discovered in 1967, with the majority of these outbreaks occurring in Africa. In 1975, the second outbreak of MVD occurred in Zimbabwe, marking the first-ever recorded outbreak of the disease in Africa^13^. Visitors to the Sinoia Caves, which were known to have insectivorous bats, developed symptoms after 8–9 days, leading to suspicion of bats or their discharge as a potential source of infection^11,14^. From 1975 to 1985, only a few cases of MVD were reported in Africa. Prior to 1998, MARV was not believed to be as fatal as the Ebola virus. However, this perception changed after two significant outbreaks occurred in the Democratic Republic of Congo between 1998 and 2000, and the first-ever outbreak in Angola between 2004 and 2005^15^. Uganda experienced four epidemics in the year 2007, 2012, 2014, and 2017, each with an incident fatality percentage ranging from 27 to 100%^11^, while between the year 1998 and 2000, the Republic of Congo reported a total of 154 cases of MVD, resulting in 128 deaths and a case-fatality rate of 83%^16^.

**Figure 1:**
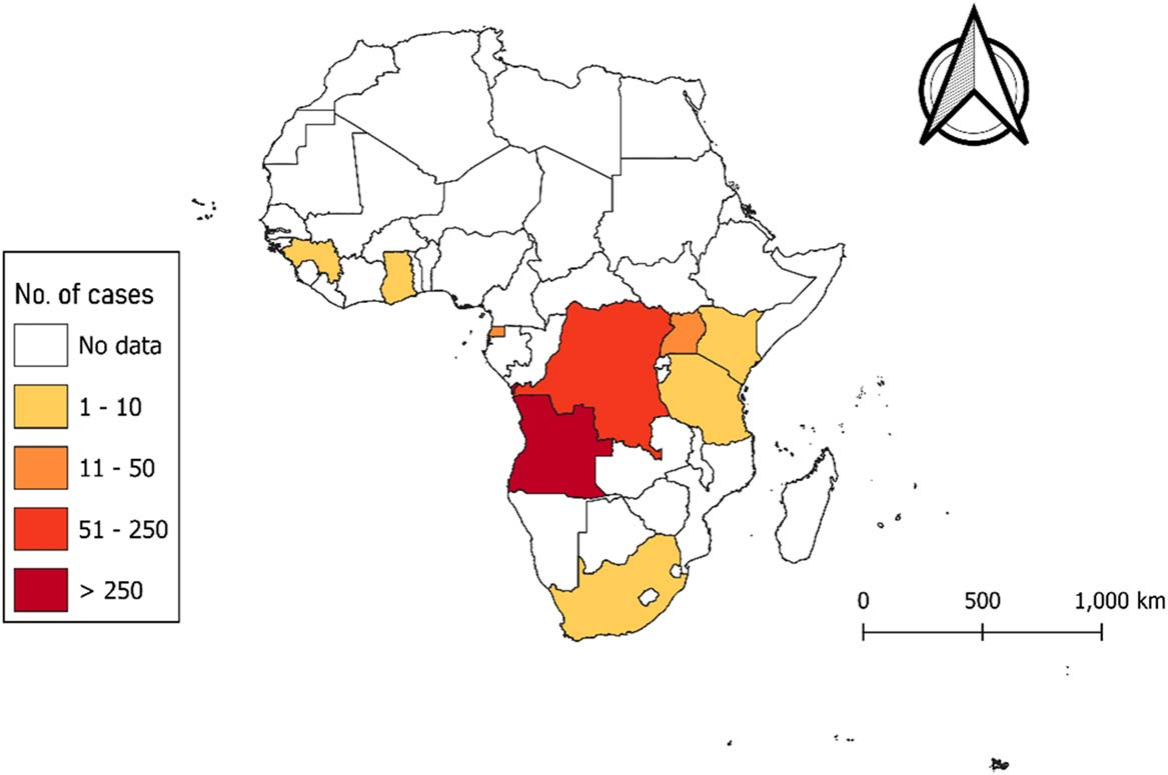
Choropleth map **s**howing the number of Marburg virus cases in the African continent between 1975 and May 2023^23,24^.

**Figure 2:**
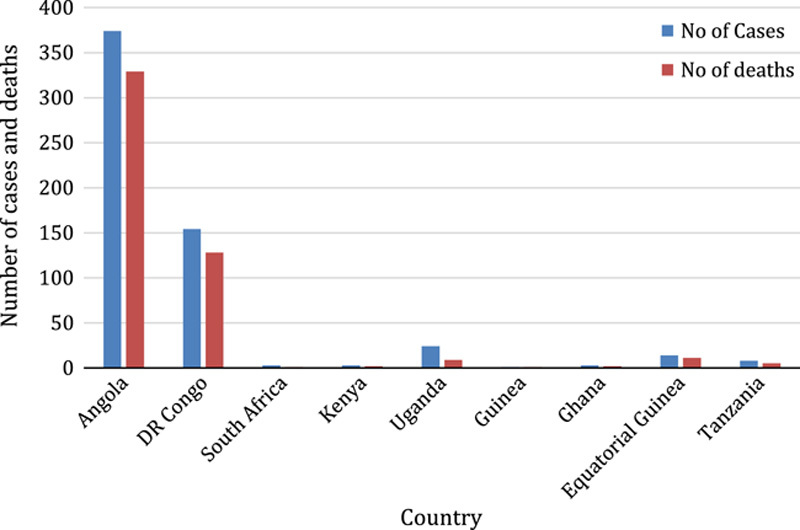
Prevalence of Marburg virus cases and Deaths by year between 1975 and May 2023 in Africa^23,24^.

**Figure 3:**
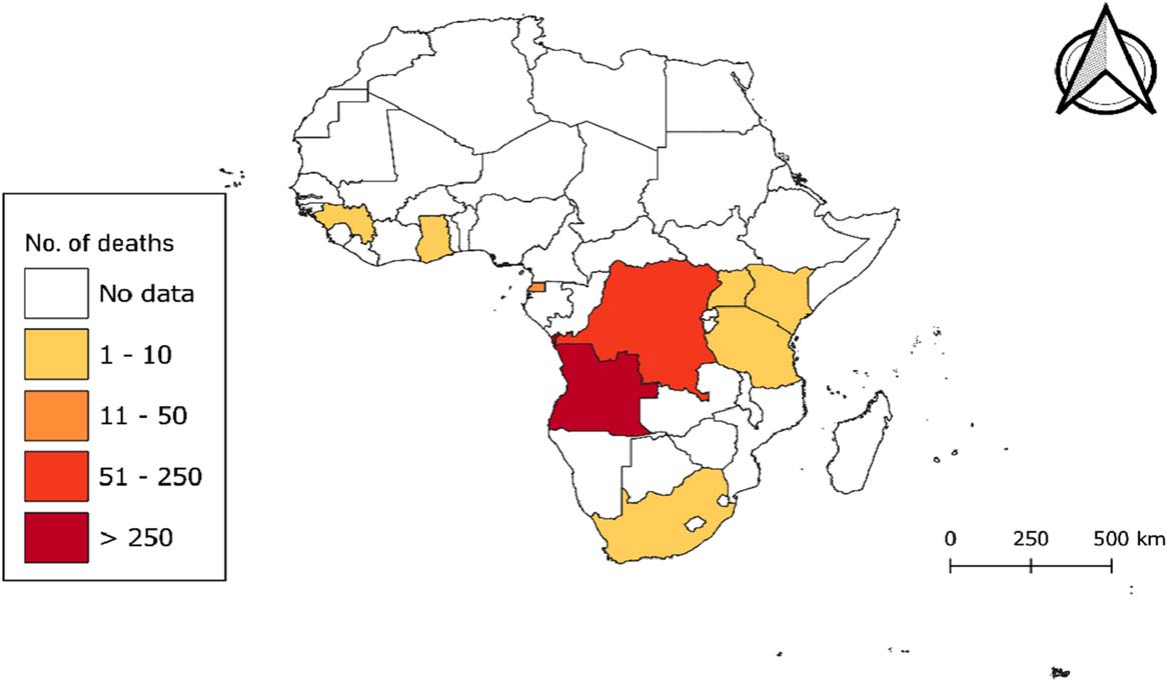
Choropleth map showing the number of Marburg virus deaths in the African continent between 1975 and May 2023^23,24^.

The first-ever MARV outbreak in West Africa was recorded in the Republic of Guinea, in August 2021^17,18^. This was followed by another outbreak in Ghana in 2022, reported as the country’s first-ever case of MARV, which resulted in the death of one male adult and the 14-month-old child by June 2022^18^. In early 2023, Equatorial Guinea and Tanzania experienced an outbreak of MARV, which was the first of its kind. In mid-February 2023, Equatorial Guinea confirmed its first-ever outbreak of MVD after conducting preliminary tests on samples from the eastern province of the country. The outbreak resulted in at least 11 deaths as at 11 April 2023 from 14 laboratory confirmed cases of which the majority of the cases were reported among females^19,20^. In Tanzania, the first outbreak was declared by the Ministry of Health on 21 March 2023 in the Bukoba district of northwestern Kagera region. The outbreak has resulted in eight confirmed cases and five deaths as of 4 April 2023, including a healthcare worker with a case-fatality rate of 63%. The remaining three patients are currently receiving treatment at designated treatment centers^21^. According to the WHO, the average case-fatality rate of MVD is 50%. However, the fatality rates in the past have varied between 24 and 88% due to differences in virus strains and the management of cases^22^ (Figs. 1–3).

## Implications of MVD in Africa

Africa has experienced a 63% increase in zoonotic diseases, 70% of which are caused by emerging deadly viruses in the last decade. This rise has added a significant burden on African healthcare systems and had an adverse impact on the overall quality of life^16^. Recent research has highlighted the negative impact on many African countries’ healthcare system due to the simultaneous occurrence of epidemics alongside numerous endemic diseases, which have further exacerbated the existing public health issues as well as overburdened the already exhausted healthcare system^16,25^. MVD is characterized by significant fluctuations in body temperature, and laboratory abnormalities such as low white and lymph cell counts, low potassium levels, and high liver enzyme levels^26^. Persons affected may experience abdominal and chest pain, prolonged bleeding time, mucosal hemorrhage, easy bruising, and impaired renal function, necessitating dialysis within the first week of illness. Severe cases of MVD can result in shock, hypotension, multiorgan failure, prostration, and social stigmatization. Gastrointestinal symptoms such as vomiting and diarrhea were also observed, leading to fluid loss, electrolyte imbalances, and acid-base disturbances^10,26^. Survivors of MVD may suffer from prolonged convalescence and various after-effects, including muscle pain, fatigue, excessive sweating, skin peeling, memory loss, and decreased sexual function. The virus can persist in the testes of nonhuman primates, live virus has been found in semen and aqueous humor samples for up to 3 months after illness^27^. In scenarios that result in death, the average time from symptom onset to death is around 9 days^14,15^.

In addition to the impact of MVD on morbidity and mortality, the condition also affects the nation’s economy, inflicts social and emotional distress on affected families, and diminishes household productivity, among other consequences^28,29^. Families are unable to participate in traditional funeral rites for their loved ones and social gatherings are prohibited^30^. Its capacity to extend across national boundaries signifies its potential to induce widespread public and global panic, disrupt international travel, and trade between countries, leading to a reduction in foreign exchange and resulting in severe socio-economic consequences^29,30^. MVD during pregnancy leads to an almost high incidence of spontaneous abortion and stillbirth^31^ while survival beyond 19 days postpartum is rare for infants born to infected mothers, indicating a very poor prognosis for congenitally infected fetuses and neonates^31^. Limited data exists regarding pediatric survival in MARV infection. However, there is a notable case of an 8-month-old infant who contracted MVD but survived. The infant’s survival was attributed to being breastfed and receiving care from the mother^32^, while a recent case of a child in Ghana infected with the highly contagious MARV passed away in August 2022^33^.

In addition, healthcare workers and family members looking after infected individuals can be at high risk of contracting the virus considering its transmission^34^. Unfortunately, there are not yet authorized antiviral therapies or vaccines available for the treatment and prevention of the disease. This is worrisome given the high mortality rate associated with the disease, as well as the possibility of future outbreaks and a multinational pandemic in other African countries^4^. In addition, inadequate coordination and preparedness for epidemics in Africa, which often results in poor disease surveillance and reporting may unfairly put its population at risk of MVD^25^. The occurrence of MVD in newly affected countries towards the ending of the COVID-19 emergency status raises significant public health challenges, not just for Africa but for the entire global community^16^.

## Multinational and global preparedness

The treatment of Marburg disease is currently supportive. Treatment in the hospital can be provided to patients, focusing on fluid and electrolyte balance, oxygenation, blood pressure, blood replacement, and treatment of co-infections^35^. There is a potential future direction in the discovery of an antiviral agent called T-705, also known as favipiravir for the treatment of MVD. T-705 has been approved for influenza treatment in Japan and has undergone phase II clinical trials for treating Ebola virus disease and currently shows promise for treating MARV infection based on laboratory and mouse model animal studies. This antiviral agent has been effective in vivo and in vitro^36,37^. Significant advancements have been made in the development of vaccines that can effectively prevent lethal filovirus infection such as Marburg and Ebola in nonhuman primates^38^. The NIAID’s Vaccine Research Center (VRC) recently developed cAd3-Marburg, an experimental vaccine for MARV. This cAd3 vaccine platform has demonstrated safety in previous clinical trials with Ebola and Sudan virus vaccines developed by the VRC^39^.

After the recent outbreak of MARV in Tanzania in March, 2023, the WHO warns that the risk of MARV is high in Africa as it continues to spread to densely populated areas, with a 90% mortality rate. At the global level, it has been stated that it is unlikely to result in a global pandemic^4^. Environmental factors including population density and mobility, healthcare infrastructure, outbreak preparedness and response capabilities, disease surveillance, public health measures, and cultural practices and burial rituals are some of the reasons for differences in cases and deaths of MVD among African countries^13,14^. African healthcare systems should be prepared and ready to efficiently tackle MVD cases in already affected and yet affected countries by investing in their healthcare system, and implementing early and coordinated measures^40^. Active disease surveillance should be massively expanded through investments in health systems to identify cases, track contacts and communities at risk, and reduce the risks of transmission to others, while appropriate infection prevention and control measures are taken to safeguard them and their contacts^16,25^, by establishing isolation centers, mobile laboratories, and a trained rapid response team^41^. Moreover, adopting the one-health concept and fostering collaboration would enhance Africa’s surveillance system and emergency response capabilities to effectively address MVD^1^. Collaboration with government and international partners, along with adequate funding will facilitate a swift and effective response^41^. There is also a need for coordinated countermeasures to strengthen the surveillance system and enable international and local contact tracing of individuals who show signs of MARV during travel or upon their return^16^. To avoid misconceptions and stigmatization that could impede contact tracing, cross-border public health education campaigns on MARV should also be carried out urgently and on a large-scale in all Africa^16^. To prepare for the possibility of both multinational and global outbreak of MARV, it is crucial to improve the training of frontline healthcare workers in the areas of timely and accurate diagnosis, effective care and management of patients with MARV^40^. Mental health and psychosocial support by professional counselors should also be offered to survivors of MVD^41^.

Establishing epidemiology, community involvement, and comprehensive surveillance initiatives, particularly in the impacted region of the continent, is of utmost importance^40^. Regulations on appropriate measures to prevent MVD such as avoiding contact with reservoir animals, ensuring thorough cooking of animal products, maintaining good hand hygiene, WHO developed guidelines for safe management and burial practices as well as options for cremation should be established in all African countries with protocols for strict adherence^17^. Only trained healthcare providers equipped with personal protective equipment should be allowed to handle potentially infected patients with Filovirus infection to prevent breaches in protocol and ensure proper care. To minimize the risk of transmission, disposable supplies should always be used for patients with Marburg infection, and the use of sharp objects should be minimized. Handling human remains should be limited, while autopsies are avoided as much as possible. It is expedient to inform state and local authorities about Marburg deaths and contaminated items, and human remains should be handled according to their guidance^42^. Once recovered from Marburg or any other Filoviruses, individuals are typically immune and noninfectious to others. However, Ebola can be present in semen for up to 3 months postrecovery, necessitating abstinence from sexual activity, including oral sex, for at least 3 months and mothers in the recovery phase should refrain from breastfeeding for at least 15 days as the virus has been found in breast milk during this period (Ebola Disease)^43^. The WHO’s country-specific recommendations should be utilized to curb MVD in areas where it is prevalent, and collaboration between the government and stakeholders is necessary to swiftly contain MARV outbreaks especially during co-morbidities currently experienced to avoid collapsing of the already fragile healthcare systems^16,17^. Investing in research and development is crucial for finding curative measures against the MARV. This involves supporting studies on antiviral drugs, vaccines, and innovative treatments tailored to MARV infection^2^. Collaborating with local and international research institutions can advance treatment strategies and deepen understanding of the virus^44^ as well as conducting clinical trials to evaluate the safety and effectiveness of potential therapies is essential for identifying effective interventions for MARV in Africa^1,39^.

## Limitations

The study briefly mentions recent MARV outbreaks in Guinea, Ghana, Equatorial Guinea, and Tanzania. However, as there is limited data, this article did not provide detailed information on the causes, extent, or impact of these outbreaks of each affected country, limiting the understanding of their implications. Also, the study mentions the absence of widely accepted vaccines or antiviral medications for MVD and gave a brief summary of the ongoing efforts but does not delve into these efforts or potential strategies for prevention and control. Exploring current research and initiatives in this area would enhance the completeness of the study.

## Conclusion

MVD is a severe hemorrhagic fever caused by the MARV, which is transmitted directly from bats or monkeys to humans or from human to human, and is able to affect both humans and nonhuman primates. Several countries in the African region have experienced a series of MARV outbreaks in the past few years. Recently, West African countries have reported their first-ever outbreaks of MARV. The possibility of future outbreaks in other African countries is high, considering the rapid increase in emerging zoonotic diseases on the continent. These outbreaks have put additional pressure on healthcare systems in Africa being adversely impacted, affecting the overall quality of life. There is an urgent need to deploy a multifaceted health system strengthening approach for the prevention and curative of MARV, and better respond to its future outbreaks and other viruses with an epidemic potential in Africa^44^.

## Ethical approval

Ethical clearance was not required because we did not deal with human or animal data.

## Consent

None.

## Sources of funding

None.

## Author contributions

O.J.O., E.M., N.O.O., H.A.O., A.A.F., O.B., O.G., J.J.L., and D.E.L.P. III: conceptualization; O.J.O., E.M., N.O.O., O.G., J.J.L., and D.E.L.P. III: methodology; H.A.O., J.J.L., O.J.O., E.M., and O.B.: validation; O.J.O., E.M., N.O.O., O.B., H.A.O., A.A.F., O.G., and D.E.L.P. III: investigation; N.O.O., E.M., J.J.L., O.B.: resources; O.J.O., E.M., A.A.F., H.A.O., J.J.L., and D.E.L.P. III: data curation; O.J.O., E.M., N.O.O., H.A.O., A.A.F., O.B., O.G., J.J.L., and D.E.L.P III: writing – original draft; O.J.O., E.M., N.O.O., H.A.O., A.A.F., O.B., O.G., J.J.L., and D.E.L.P. III: writing – review and editing; O.J.O., E.M., N.O.O., J.J.L., O.B., H.A.O., A.A.F.: visualization; O.J.O., E.M., N.O.O., O.G., J.J.L., D.E.L.P. III: supervision.

## Conflicts of interest disclosure

The authors declare no conflicts of interest.

## Research registration unique identifying number (UIN)

1. Name of the registry: not applicable.
2. Unique identifying number or registration ID: not applicable.
3. Hyperlink to your specific registration (must be publicly accessible and will be checked): not applicable.

## Guarantor

Okesanya Olalekan John.

## Data availability statement

None.

## Provenance and peer review

None.
